# Transcriptome and DNA methylation profiling during the NSN to SN transition in mouse oocytes

**DOI:** 10.1186/s12860-024-00527-3

**Published:** 2025-01-03

**Authors:** Hannah Demond, Soumen Khan, Juan Castillo-Fernandez, Courtney W. Hanna, Gavin Kelsey

**Affiliations:** 1 Epigenetics Programme, Babraham Institute, Cambridge, CB22 3AT UK; 2 Laboratory of Integrative Biology (LIBi), Centro de Excelencia en Medicina Traslacional (CEMT), Scientific and Technological Bioresource Nucleus (BIOREN), Universidad de La Frontera, Temuco, Chile; 3 Millennium Institute on Immunology and Immunotherapy, Santiago, Chile; 4 BMRC, Biomedical Research Consortium Chile, Santiago, Chile; 5 Loke Centre for Trophoblast Research, University of Cambridge, Cambridge, CB2 3EG UK; 6 Department of Physiology, Development and Neuroscience, University of Cambridge, Cambridge, CB2 3EG UK; 7 Wellcome-MRC Institute of Metabolic Science-Metabolic Research Laboratories, Cambridge, CB2 0QQ UK; 8 Present Address: Institute of Pathology, University of Cologne, Faculty of Medicine and University Hospital Cologne, Cologne, Germany

**Keywords:** Oocyte, Transcription, Chromatin, DNA methylation, Histones, scRNA-seq, scBS-seq, ChIP-seq

## Abstract

**Background:**

During the latter stages of their development, mammalian oocytes under dramatic chromatin reconfiguration, transitioning from a non-surrounded nucleolus (NSN) to a surrounded nucleolus (SN) stage, and concomitant transcriptional silencing. Although the NSN-SN transition is known to be essential for developmental competence of the oocyte, less is known about the accompanying molecular changes. Here we examine the changes in the transcriptome and DNA methylation during the NSN to SN transition in mouse oocytes.

**Results:**

To study the transcriptome and DNA methylation dynamics during the NSN to SN transition, we used single-cell (sc)M&T-seq to generate scRNA-seq and sc-bisulphite-seq (scBS-seq) data from GV oocytes classified as NSN or SN by Hoechst staining of their nuclei. Transcriptome analysis showed a lower number of detected transcripts in SN compared with NSN oocytes as well as downregulation of 576 genes, which were enriched for processes related to mRNA processing. We used the transcriptome data to generate a classifier that can infer chromatin stage in scRNA-seq datasets. The classifier was successfully tested in multiple published datasets of mouse models with a known skew in NSN: SN ratios. Analysis of the scBS-seq data showed increased DNA methylation in SN compared to NSN oocytes, which was most pronounced in regions with intermediate levels of methylation. Overlap with chromatin immunoprecipitation and sequencing (ChIP-seq) data for the histone modifications H3K36me3, H3K4me3 and H3K27me3 showed that regions gaining methylation in SN oocytes are enriched for overlapping H3K36me3 and H3K27me3, which is an unusual combination, as these marks do not typically coincide.

**Conclusions:**

We characterise the transcriptome and DNA methylation changes accompanying the NSN-SN transition in mouse oocytes. We develop a classifier that can be used to infer chromatin status in single-cell or bulk RNA-seq data, enabling identification of altered chromatin transition in genetic knock-outs, and a quality control to identify skewed NSN-SN proportions that could otherwise confound differential gene expression analysis. We identify late-methylating regions in SN oocytes that are associated with an unusual combination of chromatin modifications, which may be regions with high chromatin plasticity and transitioning between H3K27me3 and H3K36me3, or reflect heterogeneity on a single-cell level.

**Supplementary Information:**

The online version contains supplementary material available at 10.1186/s12860-024-00527-3.

## Background

Oogenesis is the process of female gamete formation, which encompasses several stages necessary to prepare an oocyte for fertilisation. Beginning during foetal life, oogonium enter meiosis and become packaged into primary follicles. After birth, the vast majority of oocytes undergo apoptosis, while the remaining progress through oocyte growth and maturation within a developing follicle, eventually leading to cyclic ovulation of a select number of these mature oocytes. During the final stages of oocyte maturation, the cell nucleus of the fully-grown mouse oocyte, also termed germinal vesicle (GV) oocyte, undergoes structural conformation changes as it transitions from a non-surrounded nucleolus (NSN) to a surrounded nucleolus (SN) type [1–3]. The NSN to SN transition is essential for the developmental competence of the oocyte and has been explored using transgenic mouse models and microscopy techniques. However, less is known about the molecular changes linked to this transition due to the difficulty in collecting these cells.

The architecture of the NSN nucleus is characterized by diffuse chromatin with a few chromatin-dense puncti representing constitutive heterochromatin [4], while the SN chromatin is condensed and forms a ring-like structure around the nucleolus. In mice, the ratio of NSN to SN oocytes shifts throughout a female’s reproductive lifespan: in newborn mice (1 week) all oocytes are at the NSN state, the proportion of SN oocytes found in an ovary increases when mice reach sexually maturity at about 4 weeks (~50% SN) and continues to increase until in aged mice (> 56 weeks) almost all GV oocytes (up to 90%) are at the SN state [3]. A similar transition has also been described for humans and other mammals [5–9].

The transition from an NSN to SN state is also marked by a number of nuclear and cytoplasmic changes, including transcriptional silencing [10, 11], the loss of higher order chromatin structure [12], and changes in morphology, localization and/or abundance of microtubules, mitochondria, the Golgi apparatus, cytoplasmic lattices and lipid droplets [13–15]. Both NSN and SN oocytes can resume meiosis and have been fertilized in vitro [16, 17]; however, NSN exhibit lower ovulation rates and developmental potential, with embryos arresting at the two-cell stage [16–20].

Before complete transcriptional silencing in the SN oocyte, the growing NSN oocyte accumulates large amounts of transcripts and proteins that are stored in the cytoplasm to support the final stages of meiosis, ovulation, fertilization and the maternal-to-zygote transition [10, 21–23]. Transcription is resumed only after fertilization during embryonic genome activation, which in mice occurs at the two-cell stage [23, 24]. This period of transcriptional silencing is crucial for meiotic and developmental competence of oocytes [10, 16, 18, 25]. Studies have shown that transcriptional silencing and the NSN to SN transition, although occurring concurrently, can be uncoupled and therefore seem to be independently regulated [2, 25–27]. Nevertheless, inhibiting transcription promotes the conversion of NSN to SN oocytes [16]. To analyse the changes in transcript abundance occurring in this phase, previous studies used microarray and bulk RNA-sequencing (RNA-seq) [15, 28]. While microarray analysis revealed only very subtle changes in expressed transcript abundances, RNA-seq was able to identify distinct signatures of NSN and SN oocytes, with SN oocytes exhibiting changes in abundance of transcripts associated with metabolic pathways, meiosis and preimplantation development. However, a recent study using published datasets reported that single-cell RNA-seq of GV oocytes may be superior to bulk analysis in providing greater reproducibility and detecting more differentially abundant transcripts [29].

Oogenesis is accompanied by dramatic changes in epigenetic marks including increases in histone 3 lysine 9 di- and tri-methylation (H3K9me2/3), H3K4me3 and DNA methylation [26, 30, 31]. Evidence from transgenic mouse models suggests that epigenetic dynamics may be regulating the NSN to SN transition and transcriptional silencing. For instance, the mRNA decay activator ZFP36L2 has recently been identified as a key factor in transcriptional silencing by promoting high histone methylation through degradation of H3K9 and H3K4 demethylases [27]. Furthermore, loss of histone modifiers such as the histone deacetylases HDAC1 and HDAC2 and the H3K9me1/2 methyltransferases EHMT2 (G9A) and EHMT1 (GLP) have been associated with impaired SN formation [32–34]. DNA methylation changes during the NSN to SN transition have not been studied in detail, although immunofluorescence analysis has indicated a global increase between NSN and SN oocytes [14, 30]. DNA methylation is erased in the germline during embryonic development and is re-set in the growing oocyte in a unique pattern [31]. Most *de novo* DNA methylation occurs at actively transcribed genes, probably through the recruitment of DNMT3A and DNMT3L to sites with H3K36me3 [31, 35]. While most of the *de novo* DNA methylation is completed in the fully-grown GV oocyte, there are differences with MII oocytes [14, 36]. These may reflect a shift in proportion of oocyte types, as MII oocytes will consist mainly of former SN oocytes, whereas fully-grown GV oocytes, usually collected from relatively young mice between 3 and 9 weeks, comprise a mixture of NSN and SN oocytes. It is not clear how methylation at the final stages of oocyte maturation is regulated and how its pattern and regulation differ from the early stages of oocyte growth.

Single-cell (sc) multi-omics studies allow interrogation of the interplay between transcriptomic and epigenomic changes in rare cell populations. In the current study, we aimed to analyse the coupling between the transcriptome and DNA methylation changes during the NSN to SN transition, using scM&T-seq [37]. This allowed us to identify differentially expressed genes between NSN and SN oocytes and to generate a classifier to infer chromatin status in datasets of oocyte single-cell and bulk RNA-seq from transgenic studies. Furthermore, using integrative analysis of DNA methylation and histone modifications, we were able to link sites gaining methylation in the SN oocyte to sites jointly enriched in H3K36me3 and H3K27me3, which is unusual as these marks are commonly not in the same regions and are opposingly associated with methylated DNA.

## Methods

### Animal housing

Mice used in this study were bred and maintained in the Babraham Institute Biological Support Unit. Ambient temperature was ~ 19–21 °C and relative humidity 52%. Lighting was provided on a 12 h light: 12 h dark cycle including 15 min ‘dawn’ and ‘dusk’ periods of subdued lighting. After weaning, mice were transferred to individually ventilated cages with 1–5 mice per cage. Mice were fed CRM (P) VP diet (Special Diet Services) *ad libitum* and received seeds (e.g., sunflower, millet) at the time of cage cleaning as part of their environmental enrichment. For collection of tissues (ovaries), mice were sacrificed by cervical dislocation, an approved method under Schedule 1 of the UK Animals (Scientific Procedures) Act 1986.

### Oocyte collection

A total of 37 fully-grown GV oocytes were mechanically dissected from the ovaries of two 12-week-old C57Bl6/Babr mice in M2 medium (Sigma-Aldrich, M7167). After removing all cumulus cells, oocytes were placed in M2 with Hoechst (5µM, Abcam, 33343) and incubated for 10 min at room temperature. Oocytes were washed in PBS and NSN/SN stage was scored on a Zeiss LSM780 confocal microscope. Absence of a ring around the nucleolus was counted as “NSN”, a partial ring as “intermediate” and a full ring “SN”. After scoring, oocytes were frozen individually in 5 µl RLT Plus buffer (Qiagen) and stored at -80 °C until further use.

### Single-cell M&T-sequencing

Single-cell RNA-seq and single-cell (sc) BS-seq libraries were prepared as described in [40]. Briefly, DNA and polyadenylated mRNA from individual oocytes were physically separated using poly-dT bound magnetic beads according to the G&T protocol published by Angermueller et al. [37]. Bead-bound mRNA was transcribed into cDNA using SuperScript II (Invitrogen) and Template-Switching Oligo primers (TSO, Eurogentec), followed by amplification of cDNA with the 2x KAPA HiFi HotStart ReadyMix (Roche) and ISPCR primers [57, 58]. Libraries were prepared from 100 to 400 pg of cDNA using the Nextera XT Kit (Illumina), per the manufacturer’s instructions but with one-fifth volumes. scBS-seq libraries were prepared as based on the protocols published by [37, 44], with minor changes as described in [40].

Libraries were sequenced on an Illumina NextSeq500. Multiplexed scRNA-seq libraries were sequenced on one MidOutput lane to an average of 3.5 million single-end reads of 150 bp read-length. Multiplexed scBS-seq libraries were sequenced in one HighOutput lane to an average of 19 million 75 bp paired-end reads (Supplementary Table 1).

### ChIP-sequencing

Ultra-low input native ChIP-sequencing was performed on pooled GV oocytes (~ 280 oocytes per replicate) from 25-day old C57BL6/Babr mice, as previously described [48]. To test the optimal antibody: chromatin ratio, an antibody titration was performed: 250ng, 125ng and 62.5ng of anti-H3K36me3 antibody on three replicates (Diagenode C15410192) was used. Libraries were QC-ed and quantified using Kapa qPCR and an Agilent Bioanalyser. Yields were the best for 250ng, so another replicate was generated using these conditions. Library yield was too low for 62.5ng so this sample was excluded, leaving a total of three replicates (replicate 1 = 250ng, replicate 2 = 125ng, and replicate 3 = 250ng). Multiplexed ChIP-seq libraries were sequenced on the Illumina NextSeq500 in High Output 75 bp single-end mode to an average depth of 32 million reads.

### Publically available data

Raw sequencing reads of scRNA-seq and scBS-seq datasets from young and aged GV oocytes were obtained from the Gene Expression Omnibus GEO, under accession code GSE154370. MII scBS-seq data were downloaded using accession code GSE56879. Accession codes for RNA-seq data sets used to test the NSN-SN classifier are listed in Table 1. H3K4me3 and H3K27me3 ChIP-seq data of GV oocytes were obtained from GSE93941.

### Library mapping and trimming

Raw FASTQ sequence files were quality trimmed and adaptor trimmed with TrimGalore! v.0.6.10 (https://www.bioinformatics.babraham.ac.uk/projects/trim_galore/) using default parameters. For sc-BS-seq, 6 bp were trimmed from both 5’ and 3’ end of the sequencing reads in single-end mode. All datasets were mapped against the mouse GRCm38 genome assembly. RNA-seq data were mapped using HISAT2 v.2.1.0 [59] (http://daehwankimlab.github.io/hisat2/main/). Mapping and methylation calling of bisulphite sequencing data were performed with Bismark v0.23.1 in single-end and non-directional mode, followed by deduplication (deduplicate_bismark) and methylation calling (bismark_methylation_extractor) [60](https://www.bioinformatics.babraham.ac.uk/projects/bismark/). ChIP-seq data were mapped with Bowtie v2.2.9 [61] (https://bowtie-bio.sourceforge.net/bowtie2/index.shtml).

### Single-cell RNA-seq analysis

Sequencing libraries were prepared for 37 samples (13 NSN, 2 Intermediate, 22 SN) and FASTQ files generated. Initial quality control was performed using FASTQC (https://www.bioinformatics.babraham.ac.uk/projects/fastqc/) and sequencing datasets with at least 1 million reads were considered for further analysis. Quantification for gene expression was performed using features derived from mouse oocyte transcriptome [39] using Seqmonk v1.48.1 (https://www.bioinformatics.babraham.ac.uk/projects/seqmonk). Two oocytes were considered as outliers based on clustering analysis and were excluded. In total, 16 SN and 9 NSN oocytes were taken forward for downstream analyses.

Differential gene expression analysis was performed using raw counts generated for the NSN and SN oocytes using DESeq2 (padj < 0.05) [62]. This list of differentially expressed transcripts was compared to the previously available list of differential genes [28] for NSN vs SN oocytes and common candidates identified (total 199 transcripts). Further, with transcripts ordered based on adjusted p values, 65 downregulated transcripts and 35 upregulated transcripts (NSN vs SN) were selected to form the “100 transcript classifier”. We also trialled 75-gene, 50-gene and 25-gene classifiers (based on both fold-change and FDR), as well as a 199-gene classifier (based on the overlap between our DEGs and those identified in ref. 28), but none performed as robustly as the 100-gene classifier. The classifier was used for generating the ‘NSN-SN classifier matrix’, a data-frame containing the normalized counts of the classifier genes for NSN and SN oocytes. The ‘NSN-SN classifier matrix’ was further used to perform clustering analysis as well as generate heatmaps using the pheatmap software from R (v1.0.12) (Kolde R (2019). pheatmap: Pretty Heatmaps._R package version 1.0.12). To use the classifier with published datasets, DESeq2 was used to generate normalized raw counts and a normalized count matrix was generated for classifier transcripts for each dataset. The matrix was further combined with the ‘NSN-SN classifier matrix’ followed by PCA and UMAP (v0.2.10.0) (Konopka T (2023). _umap: Uniform Manifold Approximation and Projection_. R package version 0.2.10.0) analyses.

### Single-cell BS-seq analysis

Single-cell BS-seq libraries prepared from a total of 35 oocytes (13 NSN and 22 SN) were obtained. In addition, we used the data of 42 young oocytes from Castillo-Fernandez et al. [40] and inferred the chromatin state using the classifier pipeline and UMAP and PCA clustering as described above (Fig. 2B). Young oocytes, with a clear clustering with either the NSN or SN oocytes, were taken ahead for methylation analyses (total 18 oocytes with 7 NSN and 11 SN). Libraries of these 53 total oocytes were further filtered and only those with > 10% mapping efficiency and covering more than 500,000 CpGs were retained for downstream analysis. Additionally, two samples which displayed very low levels of non-CpG methylation and subsequent high CpG methylation (thereby indicating possible somatic cell contamination) were also discarded. A total of 40 oocytes (28 SN and 12 NSN) were selected for downstream analysis.

Quantification for methylation data between NSN and SN oocytes was performed as described in [40]. Briefly, the genome was binned into domains based on their methylation pattern in fully-grown GV oocytes: unmethylated (0–25% methylation) and methylated (70–100% methylation) domains were defined as described by [39]. Regions in between were defined as “intermediately methylated”. These were used as probes for quantification of methylation in 3 random groups of 4 oocytes each (pseudobulk groups). To reduce false-positive errors caused by sparse coverage in single-cell methylation data, 100 iterations with different combinations of oocytes were performed. Differential methylation was calculated using a logistic regression model and domains which showed more than 10% difference between NSN and SN, in at least 50% of the combinations, were considered as differentially methylated regions. LOLA [63] was used to calculate overlap of differentially methylated regions with genomic features. For comparison of DNA methylation changes to transcriptional changes, differentially expressed genes between NSN and SN oocytes (greater than 5 kb) (excluding 1 kb downstream TSS) were used as probes. As described above, a logistic regression model was used for calculating differential methylation using pseudobulk groups (3 groups of 4 oocytes each).

### ChIP-seq analysis

H3K36me3 ChIP-seq data was analyzed alongside published H3K4me3 and H3K27me3 ChIP-seq datasets from our lab [48]. Reads per kilobase million (RPKM) was quantified for 2 kb running windows (*N* = 1,362,779). Windows with < 0.04 and > 2 RPKM in 10% input controls were excluded to eliminate unmappable regions and mapping artefacts, respectively. A total of 1,186,323 valid 2 kb running windows was used for quality control assessments and all analyses. All analyses and figures were generated in SeqMonk Version 1.48.0.

2 kb windows were defined as “enriched” for a given histone modification based on an RPKM value > 1. H3K4me3-enriched (*N* = 335,417), H3K27me3-enriched (*N* = 355,572) and H3K36me3-enriched (*N* = 376,991) domains well represent the underlying data (Fig. 4A). To compare histone enrichment to DNA methylation patterns, 2 kb windows were grouped into the following categories: H3K4me3 + H3K27me3 (*N* = 106,063), H3K4me3 (*N* = 169,128), H3K27me3 (*N* = 218,973), H3K36me3 + H3K4me3 + H3K27me3 (*N* = 6,132), H3K36me3 + H3K4me3 (*N* = 54,094), H3K36me3 + H3K27me3 (*N* = 24,404), and H3K36me3 (*N* = 292,361) (Fig. 4B). 2 kb windows within each category were then filtered for those that fell within previously defined GV methylated or unmethylated domains [39], SN hyper DMRs defined in this study, or *Uhrf1* KO oocyte hypoDMRs [47]. Distributions were compared using a Chi-Square statistic.

## Results

### NSN to SN transition is associated with transcriptome changes

**Fig. 1:**
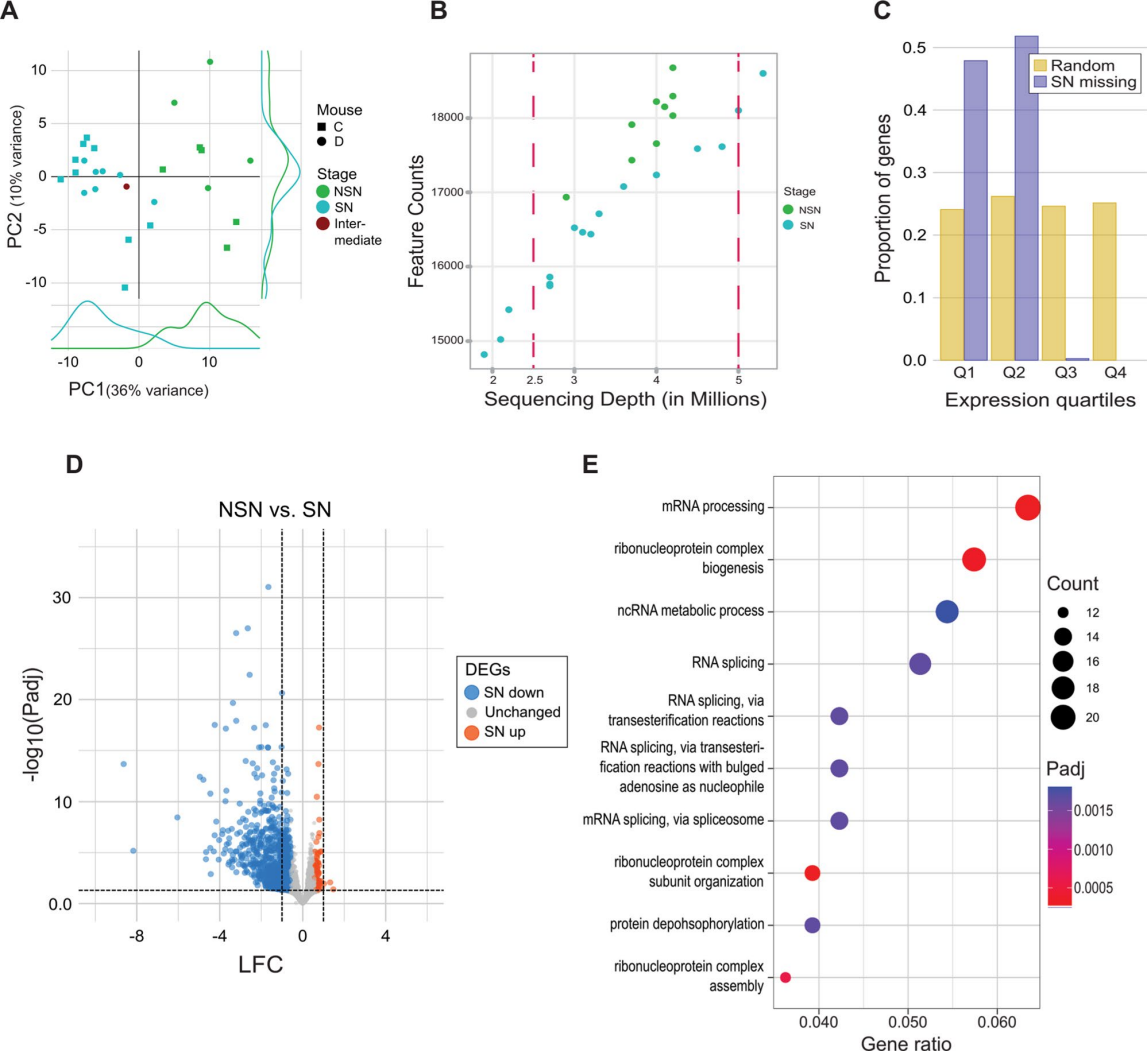
Transcriptome changes during NSN to SN transition. **A** PCA plot of scRNA-seq data of oocytes classified as NSN, SN or intermediate based on Hoechst staining. Symbol shape indicates the mouse from which the oocyte was collected. The density of NSN and SN oocyte distributions in PC1 and PC2 is also shown. The plot demonstrates that chromatin configuration is the main source of variation (PC1, 36%). **B** Scatterplot showing the total number of transcripts detected in each NSN and SN oocyte as a function of library read depth. Dots represent individual oocytes. A gene was considered to be expressed if it had at least 1 transcript count in any of the NSN or SN oocytes. SN oocytes express significantly fewer transcripts compared to NSN oocytes of comparable sequencing depths (when considering oocytes between the two dashed red lines; Wilcoxon *P* = 0.0012). **C** Barchart showing that genes not expressed in SN oocytes (SN missing) are lowly expressed in NSN oocytes (expression quartiles Q1 and Q2) compared to random genes, which are equally distributed among all NSN expression quartiles (Chi square *p* < 0.0001). Expression quartiles were determined based on all oocyte transcripts expressed in NSN oocytes. **D** Volcano plot showing differential expression between NSN and SN oocytes. Indicated are the log_2_ shrunken fold change (LFC) and the -log_10_ adjusted P-value (-log_10_ Padj). Each dot represents an oocyte transcript. Differentially expressed genes (DEGs) are highlighted in blue (down) or red (up). The dashed lines indicate the LFC > |1| and Padj < 0.05 cut-offs used to further select the DEGs for downstream analysis. **E** Dot plot showing gene ontology results for biological processes of downregulated DEGs with LFC < -1. The size of the dots indicates the number of DEGs enriched for a category and the colour signifies the adjusted P-value (Padj). The x-axis shows the ratio of DEGs enriched for a certain category

We used scM&T-seq to generate scRNA-seq and scBS-seq data from nine NSN and 18 SN GV oocytes collected from two mice and classified by Hoechst staining of their nuclei. After quality control filtering, we were left with RNA-seq data for nine NSN and 16 SN oocytes, with an average read count of 3.6 million (range: 1.9–5.3 million reads; Supplementary Table S1). Principal component analysis (PCA) resulted in distinct clustering of NSN and SN oocytes on PC1 (36% of variance), demonstrating distinct transcriptome profiles of the two GV maturation stages (Fig. 1A). As the NSN-to-SN transition coincides with transcriptional silencing [10], we analysed the total number of transcripts detected in our dataset. We observed that, when considering samples with comparable sequencing depths, SN oocytes contained on average 1,164 fewer transcript species than NSN oocytes (Fig. 1B), likely reflecting the transcript degradation associated with oocyte maturation [38]. To explore whether the absence of a specific transcript was a shared event between SN oocytes, we estimated the proportion of cells expressing each gene and observed that these events were shared across most SN oocytes (Supplementary Figure S1A). This observation suggested that heterogeneity in the transcriptome of GV oocytes is not increased after transcriptional silencing even though a reduction in expressed genes was observed. To confirm this, we conducted a differential over-dispersion test between NSN and SN oocytes. A similar number of over-dispersed transcripts was observed in both categories (NSN = 224, SN = 185), supporting the idea that both groups share similar levels of transcriptional heterogeneity (Supplementary Figure S1B). To further explore the genes no longer consistently detected in SN oocytes, we selected 382 transcripts that we characterized as “SN-missing”, based on (1) having at least 1 count in at least 7 out of 9 NSN oocytes and (2) 0 counts in at least 10 out of 16 SN oocytes (Supplementary Table S2). Gene ontology analysis of genes that annotate to SN-missing transcripts did not render any interesting results. We noted that out of 382 transcripts, 114 (29.8%) were oocyte-specific transcripts, according to our previous annotation of the mouse oocyte transcriptome [39], and a further 61 (16.0%) had no assigned gene name. Furthermore, when analysing the expression of SN-missing genes in NSN oocytes, we saw a significant bias for SN-missing transcripts to be expressed in the lowest 2 expression quartiles at the NSN stage compared to random genes (Chi square *P* < 0.0001; Fig. 1C).

Therefore, transcripts that were detected in NSN but not SN oocytes tend to be already lowly expressed in NSN oocytes. Taken together with the lack of meaningful gene ontology results and the finding that around half of these SN-missing genes do not have an assigned gene function, these results may suggest that the majority of transcript species missing in SN oocytes are not of critical functional importance for the oocyte.

Next, we used DESeq2 analysis to identify 2,347 differentially expressed transcripts (DEGs), of which 579 had a Log_2_ fold change (LFC) > |1|. Strikingly, 576 out of these 579 transcripts were downregulated (Fig. 1D, Supplementary Table S2). The vast overrepresentation of downregulated transcripts in SN oocytes is again likely to reflect the absence of active transcription in combination with transcript degradation at this stage. Gene ontology analysis of downregulated DEGs with a LFC < -1 showed enrichment for processes related to mRNA processing, including ribonucleoprotein complex assembly, RNA splicing and ncRNA metabolic process as well as protein dephosphorylation (Fig. 1E).

### Using RNA-seq data to infer chromatin configuration

**Fig. 2:**
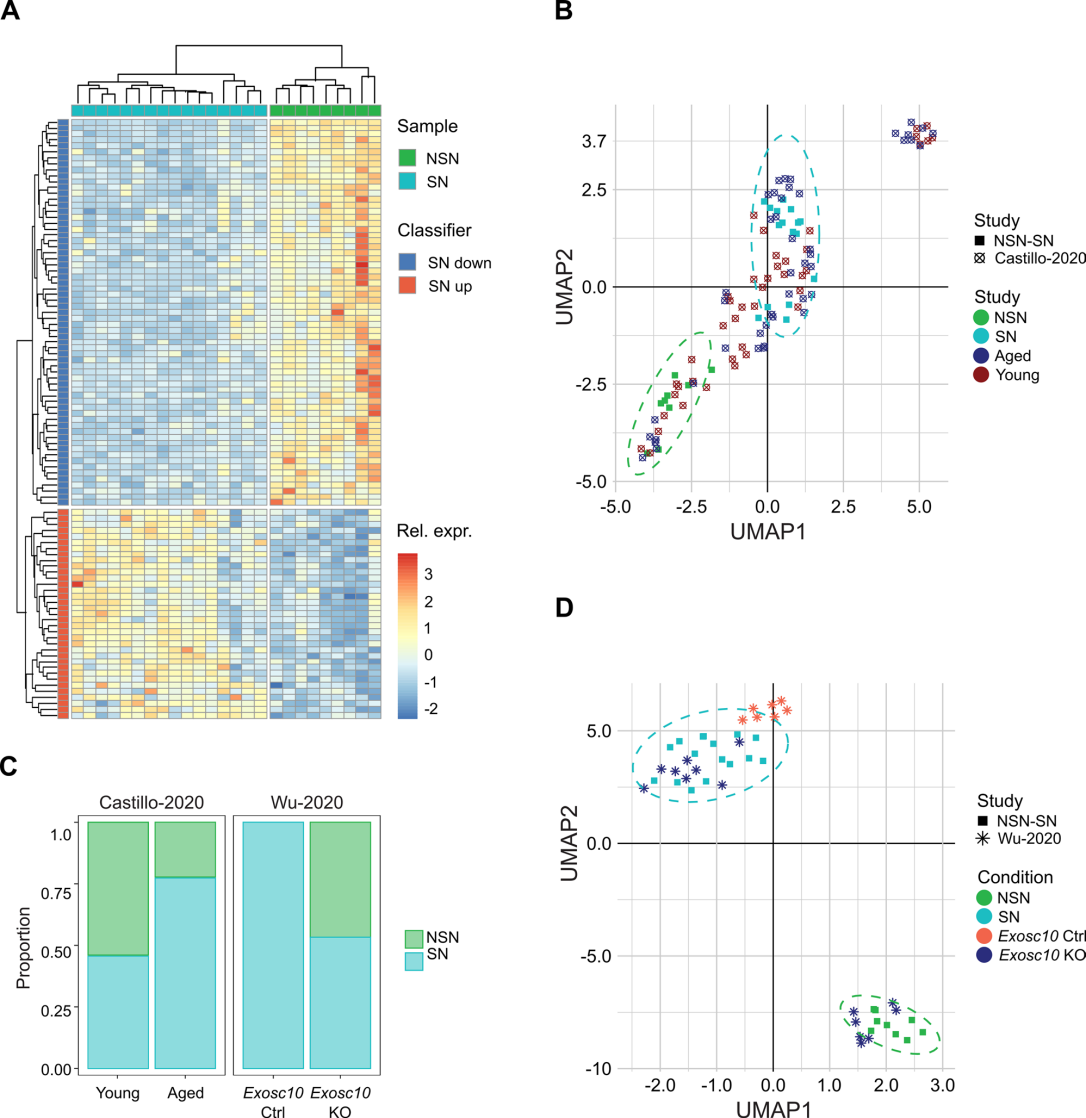
Development of a classifier to infer chromatin configuration in oocyte RNA-seq datasets. **A** Heatmap showing separate clustering of NSN and SN oocytes based on the 100 classifier genes in our NSN and SN dataset. Colour scale indicates relative expression (Z score) for each sample and classifier gene. Samples are colour-coded based on their chromatin configuration and genes are split into classifier genes that are down or upregulated in SN compared to NSN oocytes. **B**, **D** UMAP plots showing clustering based on the transcriptional profile of the 100 classifier genes of our NSN and SN oocytes together with **B** young (12 weeks) and old oocytes (> 40 weeks) from [40] and **D** *Exosc10* control and KO oocytes from [41]. The separate clusters of our NSN and SN oocytes are encircled by a dotted line. Each point indicates a separate sample. The shape of the point indicates the origin of the sample (study), whereas the colour shows the sample condition. **C** Stacked bar charts showing the proportion of NSN and SN oocytes for each genotype, based on our transcriptome classification. Aged oocytes have proportionally more SN oocytes than young oocytes, whereas *Exosc10* KO oocytes have proportionally fewer SN oocytes than *Exosc10* control oocytes

Given the strong effect that chromatin configuration has on gene expression, we attempted to generate an NSN-SN classifier to infer chromatin configuration in other RNA-seq datasets. First, we annotated the list of 2,347 differentially expressed transcripts to genes and compared them with the list of DEGs reported by Ma et al., 2013 [28] from bulk RNA-seq analysis. This resulted in 199 genes for which we extracted the associated transcripts. Next, the transcripts were divided as upregulated or downregulated in SN. For transcripts which were downregulated in SN, we chose the top 65 transcripts on the basis of FDR. We used a similar strategy for the transcripts upregulated in SN and chose the top 35 transcripts on the basis of FDR, thereby generating the 100 gene classifier (Fig. 2A, Supplementary Table S2). Application of the 100 gene classifier split our samples cleanly into NSN and SN oocytes (Fig. 2A). Surprisingly, using Uniform Manifold Approximation and Projection (UMAP) clustering analysis the Ma et al. NSN sample clustered with our SN oocytes and the Ma et al. SN sample with our NSN oocytes, independently of whether we used our 100 NSN-SN classifier genes or PCA of all 22,869 transcripts (Supplementary Figure S2A, B). As Ma et al. [28] only had one replicate sample for each group (NSN and SN), we conclude that the samples in Ma et al. have likely been mis-assigned.

To analyse whether we could use our classifier to distinguish between NSN and SN oocytes in other scRNA-seq datasets, we selected a variety of mouse models, including ageing and gene knockout (KO) models, with a known skew in their NSN-SN ratio (Table 1).

**Table 1:** scRNA-seq datasets from mouse models used to test our NSN-SN classifier

Mouse model	NSN: SN transition	Transcription	Method	GSE	Reference
Oocyte ageing	Predominant SN	Silenced in SN	scRNA-seq	GSE154370	[40]
*Exosc10* KO	Impaired		scRNA-seq	GSE141190	[41]
*Sall4* KO	Complete block	Continuous transcription	scRNA-seq	GSE84924	[42]
*Zfp36l2* KO	Normal	Continuous transcription	scRNA-seq	GSE96638	[27]
*Zcchc8* KO	Impaired	Not assessed	Bulk RNA-seq	GSE127790	[43]
*Setd2* KO	Normal	Normal	Bulk RNA-seq	GSE112835	[35]

Based on the described phenotypes for these models, we expected to see a shift in the proportion of NSN and SN oocytes compared to control oocytes. We first looked at scRNA-seq data from oocytes from young (12 weeks) and aged (> 40 weeks) mice from our previous study using the same sequencing protocol [40]. As the SN ratio increases with ageing [3], we expected to see more SN oocytes in aged oocytes compared to young oocytes. Clustering analysis based on the 100 NSN-SN classifier genes allowed us to categorize 55 out of 87 oocytes as having a clear NSN or SN signature, respectively (Fig. 2B, Supplementary Figure S2C, Supplementary Table S3). This classification indeed showed a skew in aged oocytes towards SN oocytes compared to young oocytes (Fig. 2B, C; Supplementary Figure S2C).

Next, we analysed *Exosc10* KO oocytes, which have been described to have impaired NSN to SN transition [41]. We predicted that the transcription profile of the KO oocytes would be skewed towards the NSN signature, while the corresponding wild-type (WT) oocytes would be predominantly SN. Indeed, we saw a clear difference of NSN-SN composition between *Exosc10* KO and WT oocytes (Fig. 2C, D; Supplementary Figure S3A). While all WT oocytes clustered together with our SN oocytes, the *Exosc10* KO oocytes were a mixture of NSN and SN, recapitulating the impaired, but not completely blocked, NSN to SN transition described by Wu et al. [41]. We also tested *Sall4* KO oocytes, which are described to have a complete block of the NSN to SN transition (Supplementary Figure S3B, C) [42]. Again, we saw clustering of WT oocytes with our SN oocytes, while KO oocytes cluster in proximity with our NSN oocytes.

As NSN to SN transition and transcriptional silencing can be uncoupled [2, 26], the classifier has a caveat, in that it will incorrectly identify NSN and SN state if the two processes are uncoupled. As an example, we used our classifier in *Zfp36l2* KO oocytes, in which transcriptional silencing is impaired, even though they have seemingly normal NSN to SN transition [27]. The classifier predicts *Zfp36l2* KO oocytes to be of the NSN type, while WT oocytes are of the SN type (Supplementary figure S4A, B), which reflects their impaired transcriptional state.

We also tested whether our classifier could determine NSN or SN status in bulk RNA-seq datasets. For *Zcchc8* KO oocytes, which have impaired NSN to SN transition [43], the classifier correctly identified the KO oocytes to be of the NSN type (Supplementary Figure S5A). When applied to *Set2d* KOs (Supplementary Figure S5B), which are reported to have normal NSN to SN transition and normal transcriptional silencing [35], we detected a strong divergence between the two *Setd2* KO replicates. We suspect this indicates variable proportions of NSN and SN oocytes collected for the two *Setd2* KO replicates. Overall, the classifier showed a higher accuracy/robustness when a dataset had more samples, both in single-cell and bulk datasets.

Taken together, we believe that the NSN-SN classifier based on the abundance of 100 transcripts can be a useful tool to assess a possible effect on NSN to SN transition in a variety of mouse models. However, we do recommend verifying the results by other methods, such as imaging, as transcription and chromatin conformation may be uncoupled.

### GV oocytes gain DNA methylation during the NSN to SN transition

**Fig. 3:**
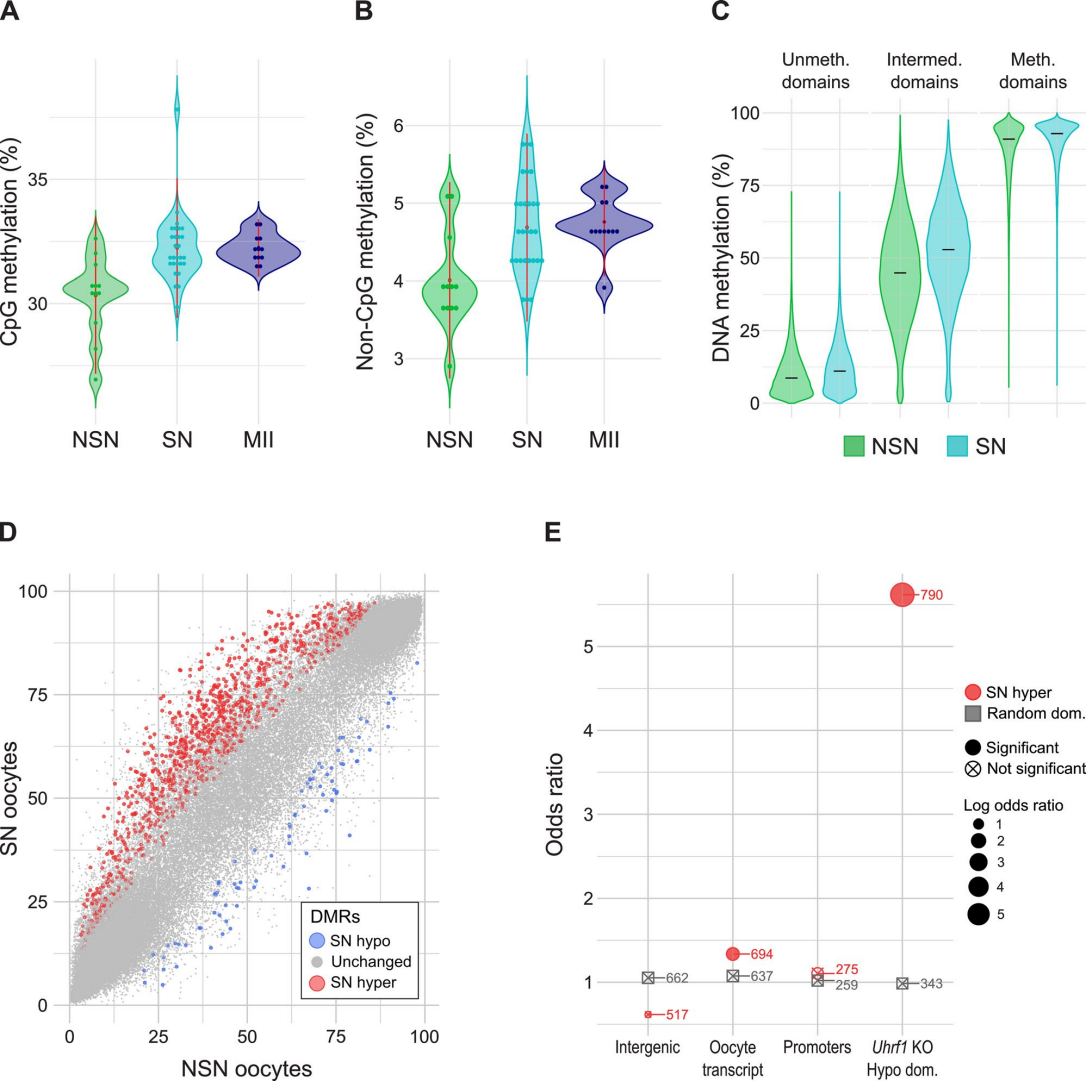
DNA methylation changes during NSN to SN transition. **A**,** B** Violin plots showing average CpG (**A**) and Non-CpG methylation (**B**) in NSN, SN and MII oocytes. **C** Violin plots comparing DNA methylation (CpG methylation) between NSN and SN oocytes in oocyte unmethylated (*N* = 52,452), intermediate (*N* = 88,103) and methylated domains (*N* = 37,272) based on published annotations from GV oocytes [39]. Intermediate (or partially) methylated domains were defined as regions covered neither by unmethylated nor methylated domains. **D** Scatterplot of DNA methylation values (%) of NSN vs. SN oocytes. Quantified were unmethylated, intermediate and methylated domains. Each dot represents one domain. Differentially methylated regions (DMRs) are highlighted in red (hypermethylated in SN) or blue (hypomethylated in SN). **E** Enrichment analysis showing the odds ratio of SN hyper DMRs (red) or random domains (grey) overlapping intergenic regions, oocyte transcripts, promoters or domains hypomethylated in *Uhrf1* KO GV oocytes. The number of domains overlapping domains with a feature is indicated. NSN and SN oocytes comprise a mixture of oocytes from the present study and oocytes from [40]. MII oocyte data is from [44]

During oocyte growth, *de novo* DNA methylation is predominantly linked to gene transcription and the deposition of H3K36me3 over expressed gene bodies. How DNA methylation levels differ between NSN and SN oocytes is unknown. To assess this, we generated scBS-seq libraries from the same oocytes used for the scRNA-seq analysis above (Supplementary Table 1). scBS-seq libraries were successfully generated for 9 NSN and 23 SN oocytes, of which only 5 NSN and 18 SN oocytes were taken ahead after QC filtering. To increase the number of oocytes for a more robust analysis, we included the scBS-seq data from young oocytes from Castillo-Fernandez et al. [40], which were made using the same protocol, and inferred their NSN and SN state with the classifier described above (Supplementary Table S3). This resulted in 12 NSN and 28 SN oocytes for downstream analysis, with coverage of between 2,198,939 and 4,817,032 CpG positions per oocyte, equivalent to ~ 10.1–22.0% of the ~ 21.9 million CpGs in the mouse genome that can be measured by BS-seq (Supplementary Table 1). As expected, we observed higher global levels of CpG methylation in SN oocytes (mean 32.3%) compared to NSN oocytes (mean 30.3%; Fig. 3A). We then extended this comparison to the next stage in oocyte development by using previously generated MII oocyte scBS-seq data [44]. Global CpG methylation in MII oocytes had similar levels to those observed in SN oocytes (Fig. 3A). Non-CpG methylation increased from a mean of 4.02% in NSN to 4.68% SN oocytes (Fig. 3B). The oocyte methylome is known to be highly bimodal, with large domains exhibiting either high or low methylation, with smaller regions in between, which may be termed “intermediately” or “partially” methylated [39, 45, 46]. We classified the genome into domains with low (< 25% methylation) and highly methylated domains (> 70%) as defined by Veselovska et al. [39]. Domains with intermediate methylation were defined as regions in between low and highly methylated domains [40]. The largest differences between NSN and SN oocytes were observed in domains with intermediate levels of CpG methylation (Fig. 3C).

Next, we sought to identify differentially methylated regions (DMRs) between NSN and SN oocytes. Because the coverage of scBS-seq libraries is low it is difficult to find domains with sufficient coverage in all samples for differential methylation analysis. Single oocytes were therefore pseudo-bulked, by randomly grouping four oocytes together and comparing three groups of four NSN and SN oocytes each in 100 iterations, as previously described [40]. By selecting only those DMRs that are shared in at least 50% of tested combinations, we were left with a robust selection of 1,146 DMRs (Fig. 3D, Supplementary Table S4). The majority of identified DMRs (1,064, 91.3%) were hypermethylated in SN oocytes. Hypermethylated DMRs were enriched for oocyte transcripts, which we used as an indicator for genes (Fig. 3E). In contrast, no significant enrichment was observed for intergenic regions or promoters. We saw the largest changes in intermediately methylated domains. Most of the SN-hypermethylated intermediately methylated domains exhibited variation in methylation level amongst individual oocytes, but were on average more methylated in SN oocytes. We also assessed the overlap with regions that are hypomethylated in *Uhrf1* KO oocytes from Maenohara et al. [47], because it was shown that loss of *Uhrf1* in growing oocytes results in hypomethylation, with intermediately methylated regions most affected [47]. We saw indeed an odds ratio of 5.62 of SN hyper DMRs for regions that lost DNA methylation in the *Uhrf1* KO oocytes (Fig. 3E).

As DNA methylation in growing oocytes is driven predominantly by active transcription, we assessed whether the expression changes observed between NSN and SN oocytes correlated with DNA methylation changes. However, there was no correlation when comparing expression changes and methylation difference, neither for the identified DEGs, nor for the DMRs (Supplementary Figure S6A, B). This is perhaps not surprising, as the transcript abundance changes observed are likely due to downstream processing of RNA and transcriptional decay, rather than changes in active transcription.

### DNA methylation changes in SN oocytes enriched for histone H3K27me3 and H3K36me3 marks

**Fig. 4:**
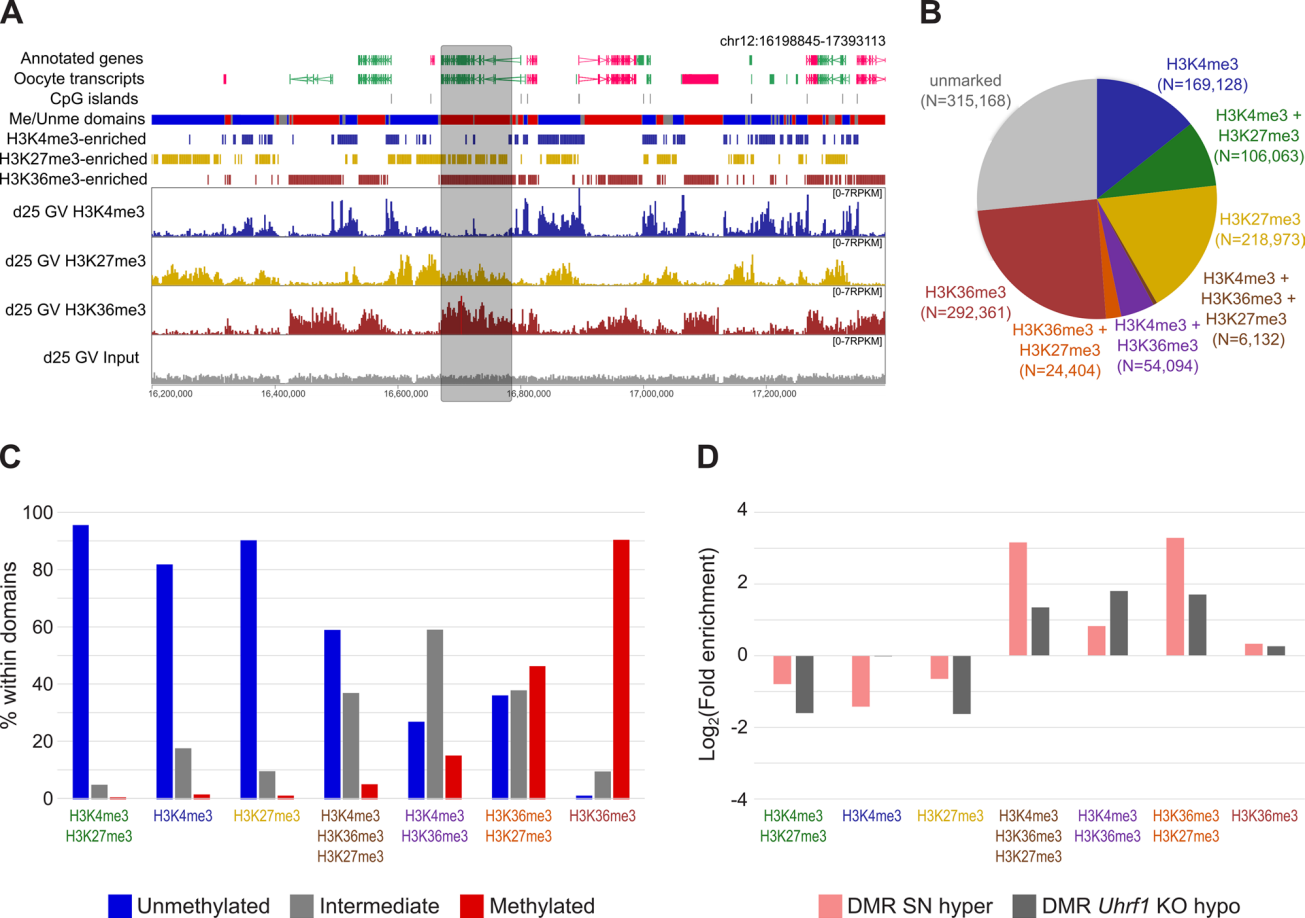
Overlap of SN differentially methylated regions with histone H3K4me3, H3K27me3 and H3K36me3 domains. **A** Genome screenshot showing the distribution of H3K4me3 (dark blue), H3K27me3 (yellow) and H3K36me3 (dark red) marks compared to input (grey) in GV oocytes. Data was quantified as RPKM for 2 kb running windows. Annotated genes and oocyte transcripts (forward in pink, reverse in green), DNA methylated (red) and unmethylated (blue) and CpG islands (grey) [64] are shown as annotation tracks above. H3K36me3 enrichment aligns well with methylated domains, as has been previously reported [51]. The grey box highlights a region with overlapping H3K27me3 and H3K36me3. **B** Pie chart showing the number of 2 kb windows (N) defined in each chromatin category, based on levels of H3K4me3, H3K27me3 and H3K36me3 in GV oocytes (Supplementary Figure S7C). **C** Barplot showing the percentage of histone-enriched 2 kb windows that fell within unmethylated, intermediate methylated or methylated domains (annotation from [39]). Chi-square statistical test (*p* < 0.00001). **D** The barplot shows the log_2_ fold enrichment for histone-marked 2 kb windows within SN hyper DMRs (*N* = 1,099) and *Uhrf1* hypo DMRs (*N* = 23,387) compared to a random set of domains (*N* = 1,100) (Chi-Square statistical test, *p* < 0.0001 and *p* < 0.0001, respectively)

Loss of DNA methylation in *Uhrf1* KO oocytes was described to be associated with untranscribed regions [47], regions expected to lack the histone posttranslational modification H3K36me3. Considering the overlap of SN hyper DMRs and *Uhrf1* KO hypo DMRs, we decided to explore the relationship between the SN DMRs and the genomic histone modification patterns. For this we performed H3K36me3 ultra-low input native ChIP-sequencing of three replicate sets containing ~ 280 GV oocytes and compared these datasets with previously published H3K4me3 and H3K27me3 datasets [48]. Replicate samples of the different histone marks showed high correlations, while correlation between different marks was much lower (Supplementary Figure S7A, B). H3K4me3, which is usually a mark of active promoters, additionally shows a non-canonical pattern of broad domains that are enriched in unmethylated, untranscribed regions in the oocyte (Fig. 4A) [46, 48, 49]. H3K27me3 is a repressive mark and usually found broadly throughout unmethylated regions (Fig. 4A) [46, 48]. H3K36me3 has been associated with actively transcribed gene bodies and overlaps regions with DNA methylation [50, 51]. Indeed, H3K36me3 enrichment aligned well with methylated domains in GV oocytes (Fig. 4A). Interestingly, we noticed regions where several histone marks were overlapping (Fig. 4B, Supplementary Figure S7C). While regions with overlapping H3K4me3 and H3K27me3 (*N* = 106,063) are well described as so-called “bivalent” domains and known to be of crucial importance for normal embryo development [52], overlap between the other marks observed here is less common. We observed 54,094 windows with H3K4me3 + H3K36me3, 24,404 windows with H3K36me3 + H3K27me3 and 6,132 windows with all three marks (H3K4me3 + H3K36me3 + H3K27me3; Fig. 4B). Supporting that these regions are co-marked, in contrast to regions with H3K36me3 alone that are almost exclusively fully methylated, these regions despite also having H3K36me3 were only partially methylated (Fig. 4C). These findings suggest that the co-occurrence of H3K4me3 and/or H3K27me3 with H3K36me3 may disrupt recruitment or activity of *de novo* DNMTs or represent regions that are heterogeneously marked between individual oocytes.

When analysing the overlap of the different histone domains with our SN hyper DMRs, the DMRs appear to be especially enriched for regions with overlapping H3K36me3 + H3K27me3 and regions with all three marks (Fig. 4D). The latter, however, comprises such a small proportion of all analysed windows that the significance is unclear. In contrast, *Uhrf1* hypo DMRs are equally enriched in H3K4me3 + H3K36me3 and H3K36me3 + H3K27me3 domains, indicating that the overlap between SN hyper and *Uhrf1* KO hypo DMRs may not be fully accounted for by the same mechanism (Fig. 4D).

## Discussion

The NSN to SN transition is a crucial step in oocyte maturation and is required for the oocyte to reach its full developmental competence [53]. The molecular mechanisms involved in the change in chromatin configuration remain poorly understood. In the present study, we profiled the transcriptome and DNA methylation changes occurring at this stage and found a decrease in total transcript numbers and downregulation of 576 gene transcripts, as well as an increase in DNA methylation in SN oocytes compared to NSN oocytes.

The transcriptome changes were not surprising, as the NSN to SN transition is well known to coincide with transcriptional silencing [10]. Genes for which transcripts were no longer detectable in SN oocytes tended to be among the low expressed genes in NSN but, at the same time, their degradation was not random. Indeed, it is thought that degradation of maternal mRNAs is a selective process, with some transcripts being protected from degradation during oocyte maturation to be translated during the oocyte-to-embryo transition [23, 38]. Gene ontology analysis of downregulated genes revealed multiple processes related to mRNA processing, especially processes involved in RNA splicing and ribonucleoprotein complex organization, which further highlights the importance of post-transcriptional regulation at this stage. These processes in general seem to be highly dynamic in the growing oocyte and fertilized zygote, as zygotic genome activation is also characterized by enrichment of transcription and splicing-related genes as well as genes involved in ribosome biogenesis [54].

Genetic mouse models are a common tool to decipher the regulatory processes during oocyte growth and maturation. In the past, numerous studies have found that knocking out a certain gene can impair or block the NSN to SN transition e.g [41–43, 55]. Based on the transcriptome changes between NSN and SN oocytes we observed in our dataset, we believe that changes in the NSN to SN ratio in such mouse models may (partly) drive the transcriptional changes observed. Indeed, studying the effects of oocyte ageing in mice, we previously found that chromatin configuration was one of the main drivers of transcriptome variation between young and old oocytes [40]. We, therefore, aimed to develop a classifier based on DEGs between NSN and SN oocytes to infer chromatin configuration using the transcriptional profile. To test this classifier, which was based on the expression of 100 transcripts, we used published RNA-seq datasets from KO mouse models with known impairments in their NSN to SN transition. As hypothesized, the classifier identified a greater proportion of SN oocytes in control compared to KO oocytes in models for *Exosc10*, *Sall4* and *Zcchc8* [41–43]. Furthermore, the classifier predicted a larger proportion of SN oocytes in aged oocytes compared to young, which is in line with literature [3]. Although the classifier was based on single-cell data, it also worked for bulk RNA-seq data. It is likely though, that the single-cell data will retrieve better results, as scRNA-seq has been shown to be more consistent with higher reproducibility between independent oocyte datasets compared to bulk RNA-seq [29]. Previous studies have used the transcription profile of NSN and SN oocytes described in Ma et al. 2013 to infer chromatin status [40, 43]. Beside the fact that, based on our analysis, we believe the single NSN and SN replicates described by Ma et al. [28] to be switched, Wu [29] found that the differentially expressed genes from the Ma et al. paper do not all behave the same way in all NSN and SN oocytes when applied to single-cell libraries. The single-cell data in our present study should better capture the intra-oocyte variance, making our classifier more robust when applied in different datasets. In summary, we believe that our classifier may be a valuable tool for future studies analysing the transcriptome in the oocyte, enabling testing of whether any transcriptional changes may be driven by changes in chromatin configuration.

We would like to emphasize that the classifier should only be used as an indicator and may not always be correct. For example, we were not able to clearly classify all oocytes as NSN and SN in the dataset from Castillo-Fernandez et al. [40]. Even though the NSN to SN transition coincides with transcriptional silencing, the processes can be uncoupled [25–27], as is the case in *Zfp36l2* KO oocytes we analysed. *Zfp36l2* KO oocytes show normal NSN to SN transition but continuous transcription in SN oocytes [27]. Since our classifier is based on the transcriptional profile of NSN and SN oocytes, this means that in a case such as the *Zfp36l2* KO, our classifier will predict a shift from SN to NSN, which does not correspond to the in vivo situation in the oocytes. It is therefore important to use the classification only as an indication or measure of quality control and to further analyse the oocytes using imaging techniques if a shift in NSN: SN proportion is predicted. We believe that this type of quality control is useful, because a shift in NSN: SN proportion could lead to the identification of spurious DEGs in a WT vs. KO comparison. Furthermore, in the *Setd2* dataset we found a high variation among the replicates within a genotype, highlighting the importance of sampling consistency and having sufficient replicates, especially for bulk RNA-seq experiments.

After most DNA methylation is erased in primordial germ cells, the genome becomes remethylated during the latter stages of oocyte growth to culminate in the distinctive DNA methylation profile of the mature gamete. This is mostly completed in the fully-grown GV oocyte; however, differences between GV and MII oocytes indicate that there are regions which gain DNA methylation at a late stage [14, 36]. In our study, we saw an increase in DNA methylation between NSN and SN oocytes, with SN oocytes exhibiting similar levels of DNA methylation compared to MII oocytes. The greatest number of changes were observed in regions with intermediate levels of DNA methylation. This has also been observed in genetic oocyte KO models, such as the *Uhrf1* KO and *Ehmt1/2* KO [34, 47], which may indicate that DNA methylation at intermediate domains is more dynamic and variable. Indeed, the SN hypermethylated regions were highly enriched for regions hypomethylated in *Uhrf1* KO oocytes. In line with this, we saw that intermediately methylated regions, as well as SN hypermethylated and *Uhrf1* KO hypomethylated regions, are enriched for domains with multiple histone marks (H3K36me3 + H3K4me3, H3K36me3 + H3K27me3 or H3K36me3 + H3K4me3 + H3K27me3). Altogether, H3K36me3 was highly associated with methylated DNA and together with findings that loss of the H3K36me3 methyltransferase SETD2 leads to depletion of DNA methylation [35], supports that H3K36me3 is necessary for *de novo* DNA methyltransferase recruitment globally in the oocyte. Paradoxically, targeted mutation of the H3K36me3-binding PWWP domain in DNMT3A does not lead to impaired DNA methylation patterning at H3K36me3 marked regions in the oocyte [56]. Hence, the link between H3K36me3 and *de novo* DNA methylation in the oocyte may be an indirect mechanism, still to be fully elucidated.

In contrast to intermediately methylated and *Uhrf1* KO hypomethylated regions, SN hypermethylated regions were particularly enriched for H3K36me3 + H3K27me3, indicating a more specific mechanism. While H3K36me3 is associated with gene bodies of actively transcribed genes and DNA methylation, H3K27me3 is found in inactive regions and is mutually exclusive with DNA methylation [31]. Therefore, these two marks usually do not overlap, raising the question of whether they really overlap in the oocyte, or whether there is heterogeneity between oocytes. This could either be because these marks are less conserved at these regions, causing heterogeneity and therefore leading to a more intermediate DNA methylation pattern. It may also be that one or other mark is transient, for example a shift of H3K27me3 to H3K36me3, which would also explain the late methylation of these regions.

## Conclusions

The NSN-SN transition in mouse oocytes is accompanied by a reduction in transcriptome complexity. Down-regulated genes in SN oocytes are enriched in multiple processes related in particular to mRNA processing, highlighting the importance of post-transcriptional regulation in preparation for the oocyte-to-embryo transition. Using a set of 100 NSN: SN DEGs, we develop a classifier that can be used to infer chromatin status in single-cell or bulk RNA-seq data. The classifier performs well against published datasets, enabling identification of altered chromatin transition in genetic knock-outs; it may also be useful as a quality control to identify skewed NSN-SN proportions that could otherwise confound differential gene expression analysis. Integration of DNA methylation data with histone modification datasets suggests that H3K36me3 is universally required for *de novo* methylation in mouse oocytes; partially methylated regions are marked by H3K36me3 but in combination with other modifications that are normally anti-correlated with DNA methylation. Late-methylating regions in SN oocytes are predominantly intermediately methylated and typified by joint enrichment for H3K27me3 and H3K36me3, which may correspond to regions that are transitioning between H3K27me3 and H3K36me3, or reflect heterogeneity between oocytes.

## Electronic supplementary material

Below is the link to the electronic supplementary material.

Supplementary Material 1: Supplementary Figures S1–S7

Supplementary Material 2: Table S1: Sequencing info RNA-seq, BS-seq and H3K36me3 ChIP-seq libraries

Supplementary Material 3: Table S2: Lists of SN missing transcripts, differentially expressed transcripts (DEGs), classifier transcript list (including genome annotation, gene names, ENSEMBL IDs and expression values/LFC, p-value)

Supplementary Material 4: Table S3: NSN/SN classification of young oocytes from Castillo-Fernandez et al. 2020

Supplementary Material 5: Table S4: List of NSN-SN DMRs (including genome annotation, methylation values or difference, and hypo/hyper DMR classification)

## Data Availability

scRNA-seq and scBS-seq data of Hoechst-stained GV oocytes and H3K36me3 ChIP-seq data of GV oocytes generated in this study are deposited in the Gene Expression Omnibus (GEO) database under accession code GSE274327 (scRNA-seq; https://www.ncbi.nlm.nih.gov/geo/query/acc.cgi?acc=GSE274327); GSE274300 (scBS-seq; https://www.ncbi.nlm.nih.gov/geo/query/acc.cgi?acc=GSE274300); and GSE274329 (H3K36me3 ChIP-seq; https://www.ncbi.nlm.nih.gov/geo/query/acc.cgi?acc=GSE274329).
