# Supplementary material for: Transcriptome and DNA methylation profiling during the NSN to SN transition in mouse oocytes
Source: BMC Mol Cell Biol. 2025 Jan 3;26:2. doi: 10.1186/s12860-024-00527-3 (PMC11697814; doi:10.1186/s12860-024-00527-3)
Supplement: Supplementary file 1 — Supplementary Material 1: Supplementary Figures S1–S7 [file 12860_2024_527_MOESM1_ESM.docx]

**Supplementary Figures**


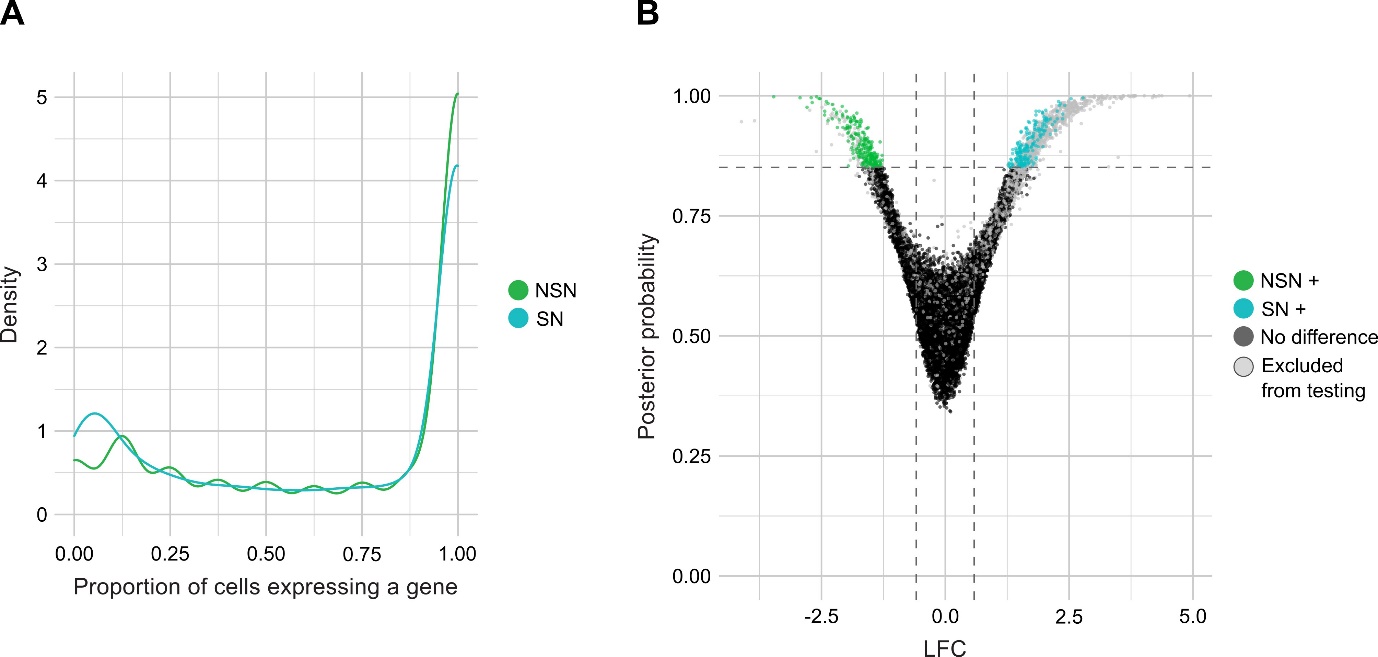


**Supplementary Figure S1: Transcriptome heterogeneity between NSN and SN oocytes. A)** Proportions of cells that express a specific gene, showing that the lack of expression of a certain gene is a shared event between SN oocytes and not random. **B)** Volcano plot showing differential over-dispersion between NSN and SN, demonstrating that heterogeneity is similar between NSN and SN oocytes.


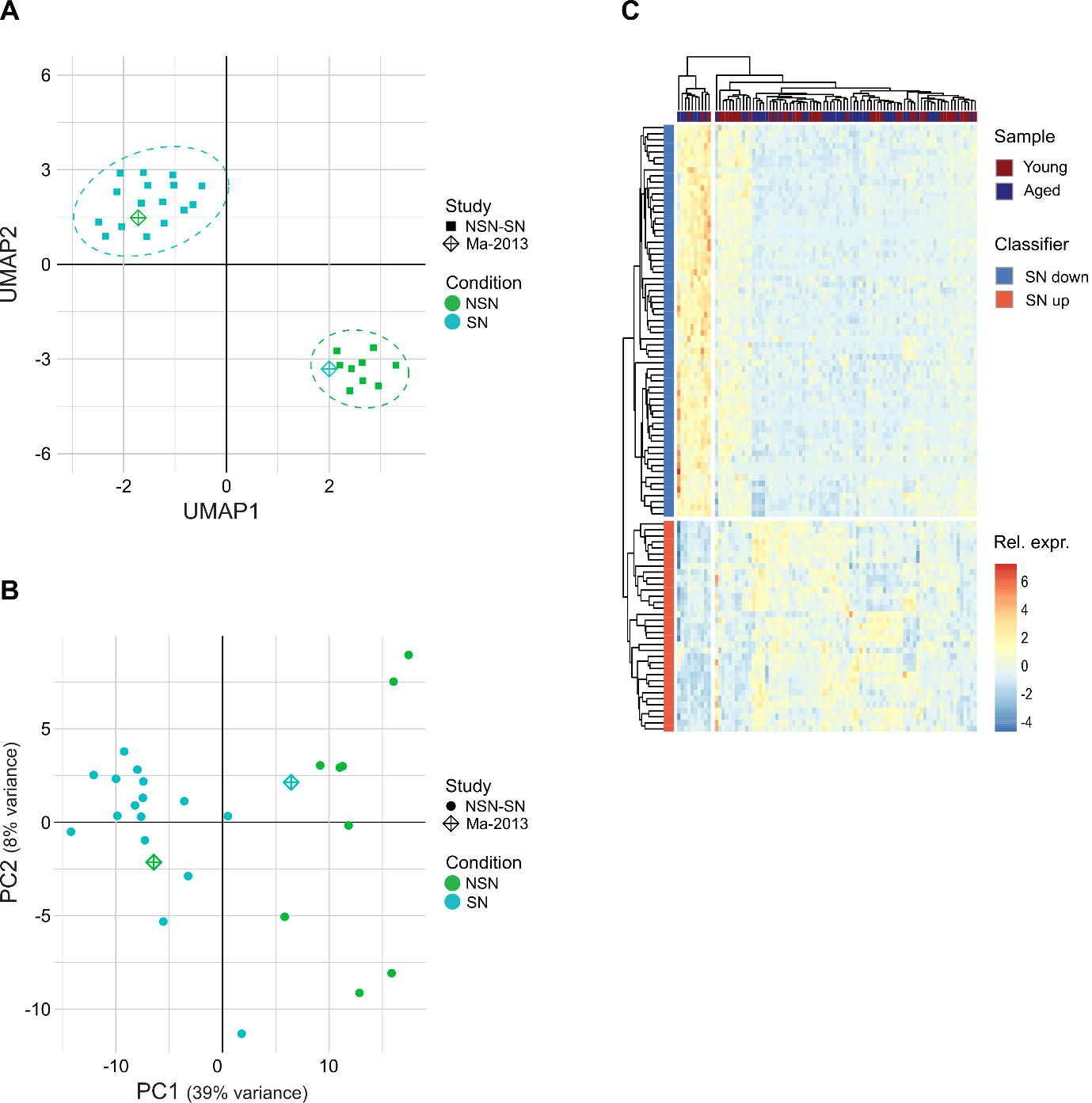


**Supplementary Figure S2: Comparison of NSN-SN classifier with Ma et al. (1) and in aged and young oocytes. A)** UMAP plot showing that the NSN sample of (1) clusters with our SN oocytes, whereas the SN sample clusters with our NSN oocytes. Clustering based on transcriptional profile of 100 NSN-SN classifier genes. The separate clusters of our NSN and SN oocytes are encircled by a dotted line. Each point indicates a separate sample. The shape of the point indicates the origin of the sample (study), whereas the colour shows the sample condition. **B)** PCA plot showing that the NSN sample of Ma et al. 2013 clusters with our SN oocytes, whereas the SN sample clusters with our NSN oocytes. Clustering based on transcriptional profile of all 22,869 transcripts. **C)** Heatmap showing separate clustering of NSN and SN oocytes based on the 100 classifier genes in young (12 weeks) and aged (>40 weeks) oocytes from Castillo-Fernandez et al. (2). Colour scale indicates relative expression (Z score) for each sample and classifier gene. Samples are colour-coded based on mouse age and genes based on the expression change of the classifier genes in SN vs. NSN genes.


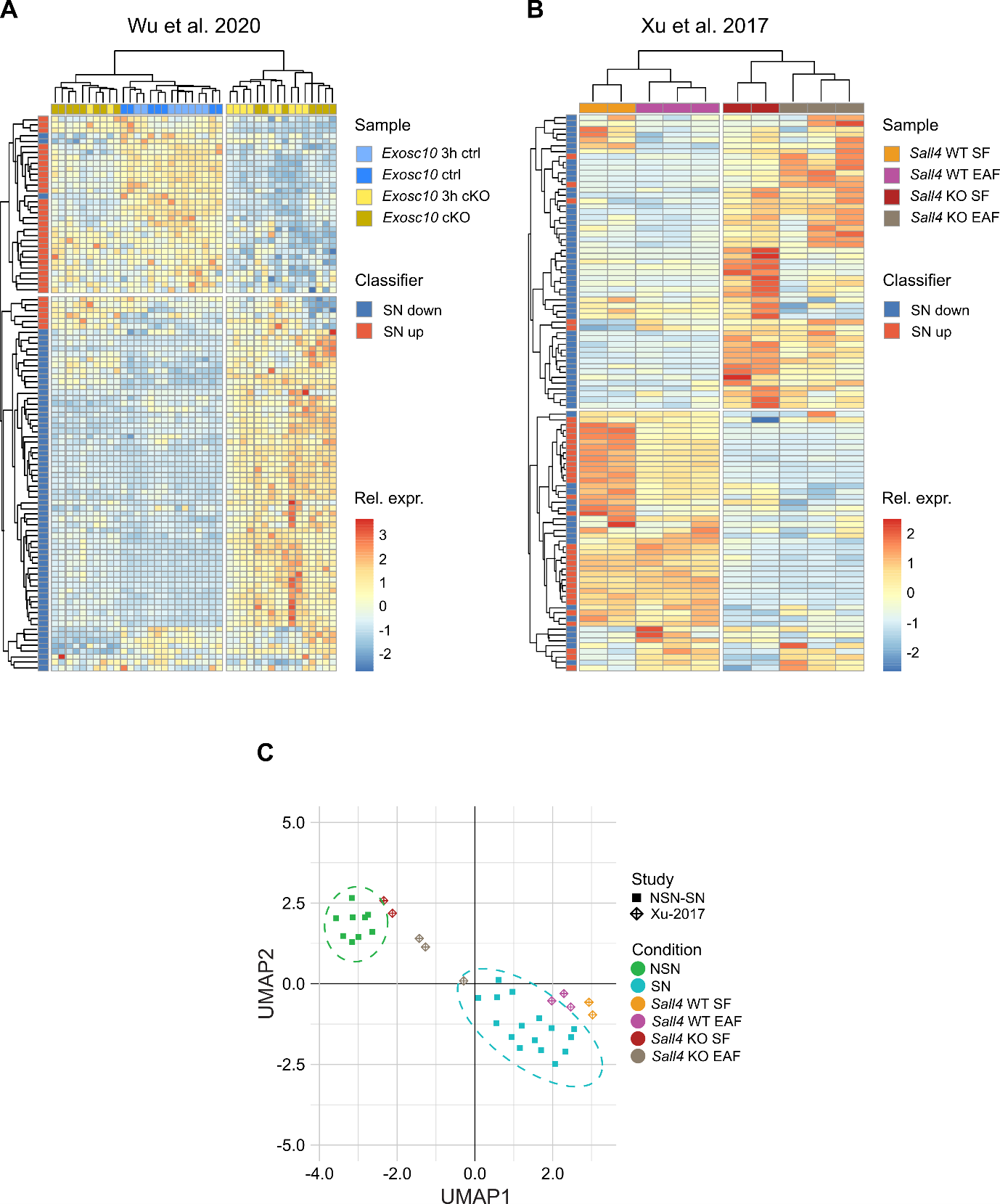


**Supplementary Figure S3: Using the NSN-SN classifier in different mouse models with impaired NSN to SN transition. A, B)** Heatmaps showing separate clustering of NSN and SN oocytes based on the 100 classifier genes in the **A)** *Exosc10* KO mouse model from Wu et al. (3)and **B)** *Sall4* KO mouse model from Xu et al. (4). Colour scale indicates relative expression (Z score) for each sample and classifier gene. Samples are colour-coded based on mouse genotype and genes based on the expression change of the classifier genes in SN vs. NSN genes. **C)** UMAP plot showing that *Sall4* WT oocytes cluster with SN oocytes, whereas *Sall4* KO oocytes cluster with NSN oocytes. Clustering is based on transcriptional profile of 100 NSN-SN classifier genes. The separate clusters of our NSN and SN oocytes are encircled by a dotted line. Each point indicates a separate sample. The shape of the point indicates the origin of the sample (study), whereas the colour shows the sample condition. In **A**, ctrl corresponds to control (*Exosc10* wildtype) and cKO to conditional (oocyte-specific) *Exosc10* knockout oocytes. In **B** and **C**, EAF corresponds to oocytes from early antral follicles; SF from secondary follicles.


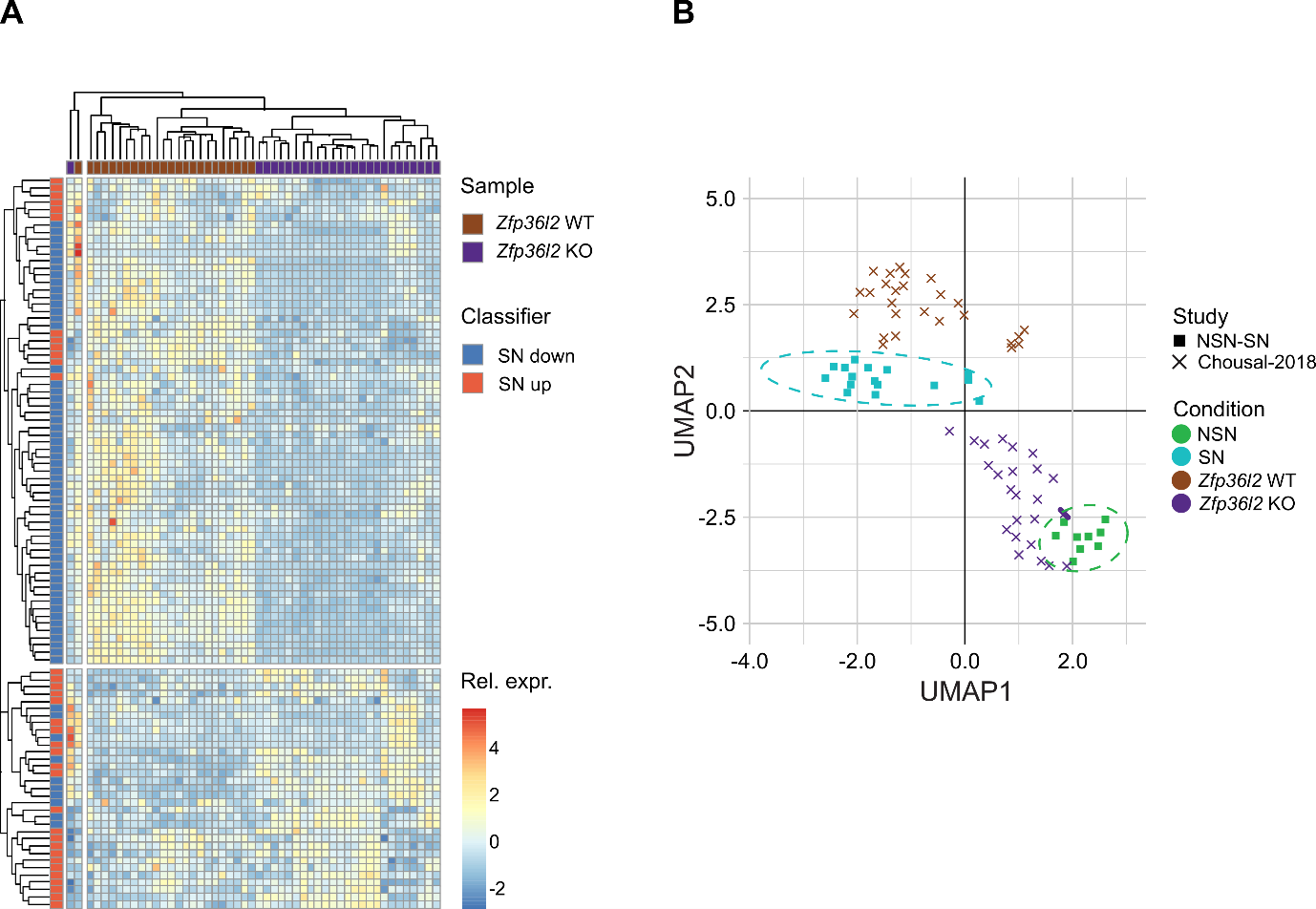


**Supplementary Figure S4:** **In *Zfp36l2* KO oocytes the NSN-SN classifier predicts impaired NSN to SN transition. A)** Heatmap showing separate clustering of NSN and SN oocytes based on the 100 classifier genes in the *Zfp36l2* KO mouse model from Chousal et al. (5). Colour scale indicates relative expression (Z score) for each sample and classifier gene. Samples are colour-coded based on mouse genotype and genes based on the expression change of the classifier genes in SN vs. NSN genes. **B)** UMAP plot showing that *Zfp36l2* WT oocytes cluster in proximity with SN oocytes, whereas *Zfp36l2* KO oocytes cluster in proximity with NSN oocytes. Clustering is based on transcriptional profile of 100 NSN-SN classifier genes. The separate clusters of our NSN and SN oocytes are encircled by a dotted line. Each point indicates a separate sample. The shape of the point indicates the origin of the sample (study), whereas the colour shows the sample condition.


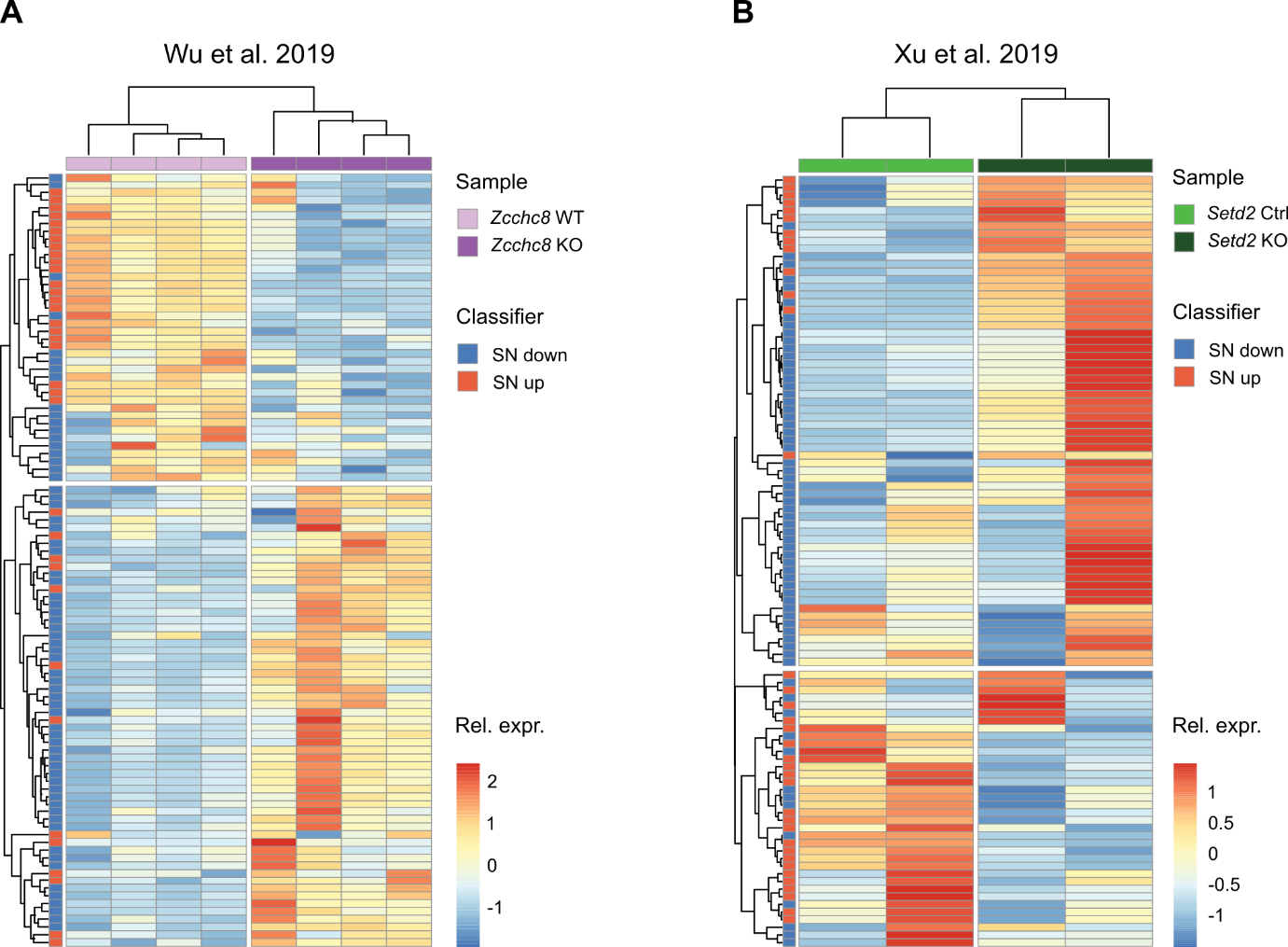


**Supplementary Figure S5: Using the NSN-SN classifier in bulk RNA-seq datasets. A, B)** Heatmap showing clustering of the 100 classifier genes in **A)** the *Zcchc8* KO mouse model from Wu et al. (6) and **B)** the *Setd2* KO mouse model from Xu et al. (7). Colour scale indicates relative expression (Z score) for each sample and classifier gene. Samples are colour-coded based on mouse genotype and genes based on the expression change of the classifier genes in SN vs. NSN genes.


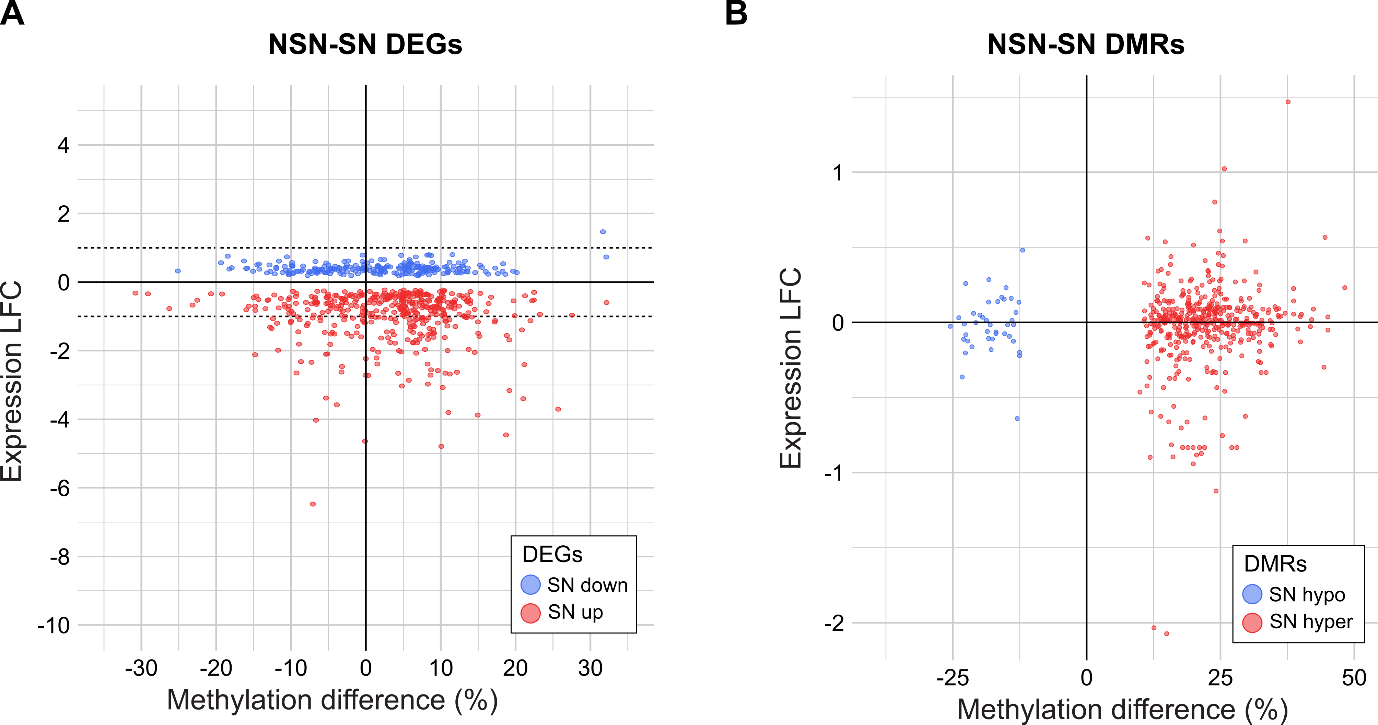


**Supplementary Figure S6: Correlation of expression and methylation changes between NSN and SN oocytes.** Scatterplots plotting Log_2_ fold change (LFC) expression changes (SN/NSN) against percentage methylation differences (SN – NSN) for differentially methylated regions (DMRs) **(A)** and differentially expressed genes (DEGs) **(B)**. Each dot represents a DMR/gene. **A)** LFC was determined for the gene closest to the DMR. **B)** Average methylation of DEGs was calculated for the gene body (1kb downstream of TSS) for DEGs with a length >5kb. No correlation regarding expression and methylation changes were observed.


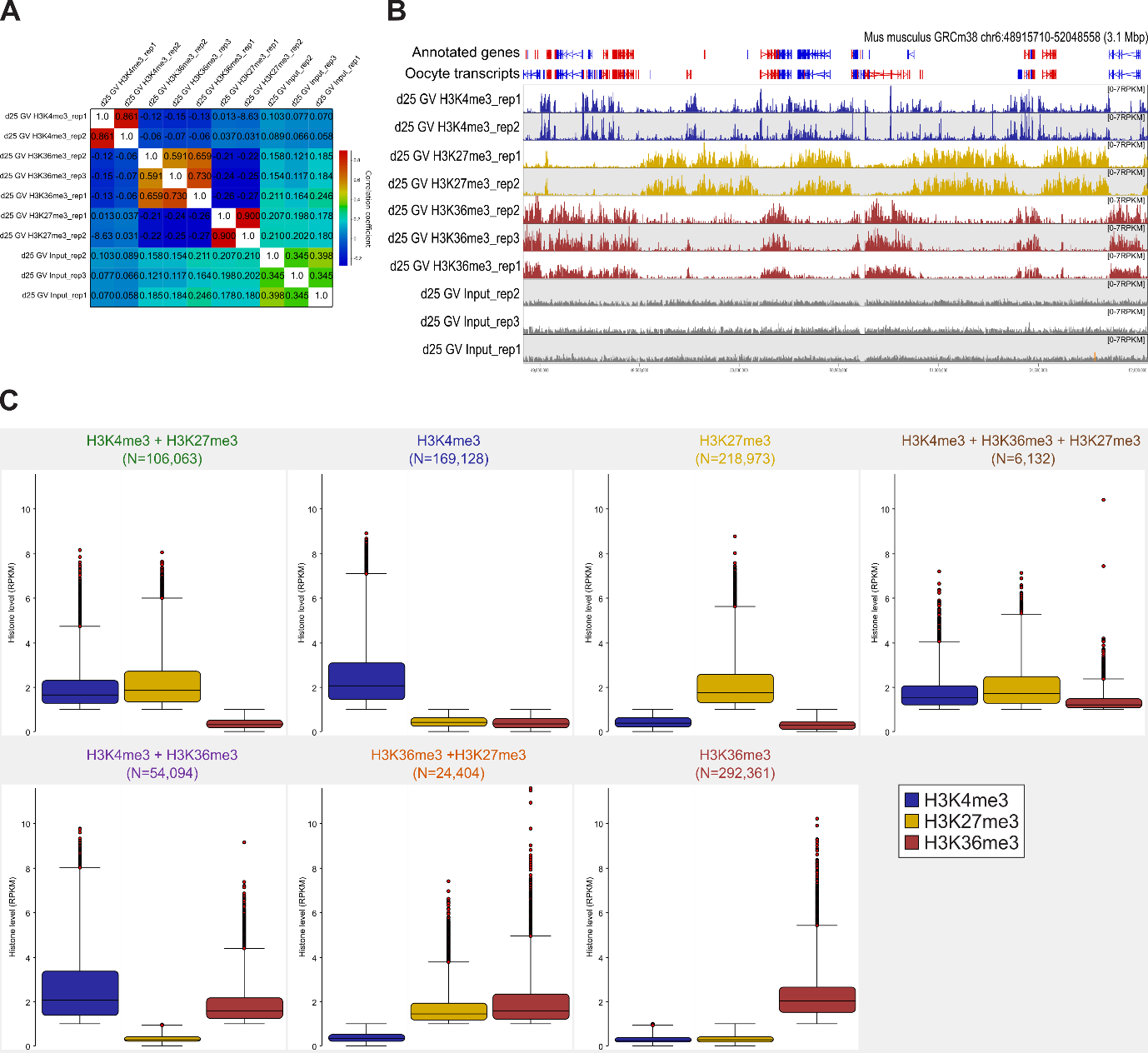


**Supplementary Figure S7: Quantification of histone marks in GV oocytes. A)** Correlation matrix between ChIP-seq replicates using 2kb windows quantified as RPKM. **B)** Genome screenshot showing the replicates of H3K4me3, H3K27me3 and H3K36me3 ChIP-seq datasets in GV oocytes. Data was quantified as RPKM for 2kb running windows. **C)** Boxplots showing the histone mark levels (RPKM) for 2kb windows categorized into: H3K4me3+H3K27me3, H3K4me3, H3K27me3, H3K36me3+H3K4me3+ H3K27me3, H3K36me3+H3K4me3, H3K36me3 +H3K27me3, and H3K36me3.

**References**

1. Ma JY, Li M, Luo YB, Song S, Tian D, Yang J, et al. Maternal factors required for oocyte developmental competence in mice: Transcriptome analysis of non-surrounded nucleolus (NSN) and surrounded nucleolus (SN) oocytes. Cell Cycle. 2013;12(12):1928–38.

2. Castillo‐Fernandez J, Herrera‐Puerta E, Demond H, Clark SJ, Hanna CW, Hemberger M, et al. Increased transcriptome variation and localised DNA methylation changes in oocytes from aged mice revealed by parallel single‐cell analysis. Aging Cell. 2020 Dec 17;19(12).

3. Wu D, Dean J. EXOSC10 sculpts the transcriptome during the growth-to-maturation transition in mouse oocytes. Nucleic Acids Res. 2020;48(10):5349–65.

4. Xu K, Chen X, Yang H, Xu Y, He Y, Wang C, et al. Maternal Sall4 is indispensable for epigenetic maturation of mouse oocytes. Journal of Biological Chemistry. 2017;292(5):1798–807.

5. Chousal JN, Cho K, Ramaiah M, Skarbrevik D, Mora-Castilla S, Stumpo DJ, et al. Chromatin Modification and Global Transcriptional Silencing in the Oocyte Mediated by the mRNA Decay Activator ZFP36L2. Dev Cell. 2018;44(3):392–402.

6. Wu Y, Liu W, Chen J, Liu S, Wang M, Yang L, et al. Nuclear Exosome Targeting Complex Core Factor Zcchc8 Regulates the Degradation of LINE1 RNA in Early Embryos and Embryonic Stem Cells. Cell Rep. 2019;29(8):2461–72.

7. Xu Q, Xiang Y, Wang Q, Wang L, Brind’Amour J, Bogutz AB, et al. SETD2 regulates the maternal epigenome, genomic imprinting and embryonic development. Nat Genet. 2019;51(5):844–56.
